# Inverted gastric adenocarcinoma of fundic gland mucosa type colliding with well differentiated adenocarcinoma: A case report

**DOI:** 10.1097/MD.0000000000007080

**Published:** 2017-06-08

**Authors:** Keitaro Takahashi, Mikihiro Fujiya, Shin Ichihara, Kentaro Moriichi, Toshikatsu Okumura

**Affiliations:** a Division of Gastroenterology and Hematology/Oncology, Department of Medicine, Asahikawa Medical University, Asahikawa; b Department of Surgical Pathology, Sapporo Kosei General Hospital, Sapporo, Japan.

**Keywords:** collision, gastric adenocarcinoma of fundic gland mucosa type, inverted growth

## Abstract

**Rationale:**

Gastric adenocarcinoma of fundic gland mucosa type (GA-FGM) is a rare tumor composed of atypical cells with differentiation toward the fundic gland as well as the foveolar epithelium. Including our case, only 9 cases of GA-FGMs were reported from 2010 to 2016.

**Concerns of the patient:**

An 87-year-old man was referred to our institution for endoscopic resection of a gastric lesion. The tumor was classified as type 0-I + IIa according to the Paris classification. Magnifying endoscopy with narrow band imaging (ME-NBI) revealed different structures of crypts and vessels among the components, illustrating the collision of 2 types of gastric cancer.

**Interventions:**

We performed endoscopic submucosal dissection and successfully removed the tumor en bloc.

**Outcomes:**

The histological findings differed markedly between the 0-I lesion and the 0-IIa lesion. The superficial part of the 0-I lesion consisted of a papillary structure, and the deeper part consisted of a tubular structure that showed inverted downward growth to the submucosal layer with the lamina muscularis mucosae. Immunohistochemically, the superficial part of the 0-I lesion was positive for MUC5AC, which had differentiated to foveolar epithelium. The deeper part was positive for pepsinogen-I and MUC6, which had differentiated to fundic gland. The 0-I lesion was diagnosed as gastric phenotype of adenocarcinoma differentiated to fundic gland mucosa with upward growth in the superficial part and downward growth in the deeper part. The 0-IIa lesion was composed of a tubular structure positive for MUC2, and it was diagnosed as an intestinal phenotype of well differentiated adenocarcinoma. The boundary was clear, and no transitional tissue was observed between the 0-I and 0-IIa lesions, suggesting that the 0-I + IIa lesion was a gastric collision tumor of GA-FGM and well differentiated adenocarcinoma.

**Lessons:**

We herein report the first case of inverted GA-FGM colliding with well differentiated adenocarcinoma. ME-NBI can be used to diagnose GA-FGM even if the lesion collides with other types of adenocarcinoma.

## 1 Introduction

Gastric adenocarcinoma of fundic gland type (GA-FG) was proposed as a new, rare variant of gastric adenocarcinoma in 2010.^[1]^ GA-FG is defined as a well differentiated adenocarcinoma with chief cell differentiation and positive staining for pepsinogen I (Pep).^[2]^ It has been reported that GA-FG accounts for 0.98% to 1.6% of gastric cancer cases,^[3,4]^ and the number of reported cases has been increasing.^[5,6]^ Recently, Tanabe et al^[7]^ reported that gastric adenocarcinoma of fundic gland mucosa type (GA-FGM) included a phenotype, showing atypical cells with differentiation toward the fundic gland as well as the foveolar epithelium. However, the endoscopic features of GA-FGM remain unclear, particularly for inverted lesions. We herein report the first case of inverted GA-FGM colliding with well differentiated adenocarcinoma.

## 2 Case report

**Figure 1:**
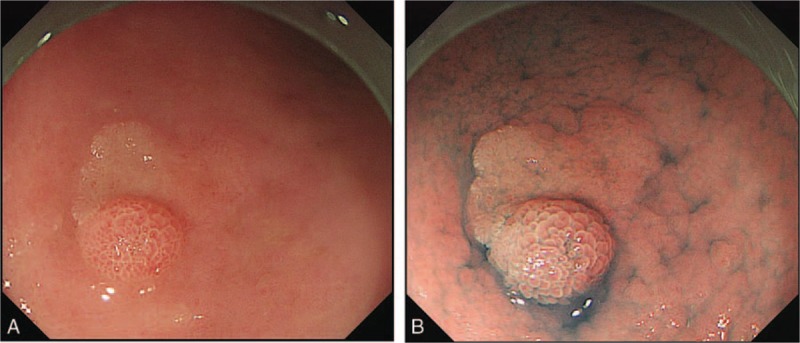
Conventional endoscopy findings. An endoscopic examination revealed a reddish, elevated lesion with faint reddish area on the great curvature of the gastric lower body, which was classified as type 0-I + IIa according to the Paris classification (A). Chromoendoscopy revealed a well demarcated line around the 0-I + IIa lesion (B).

**Figure 2:**
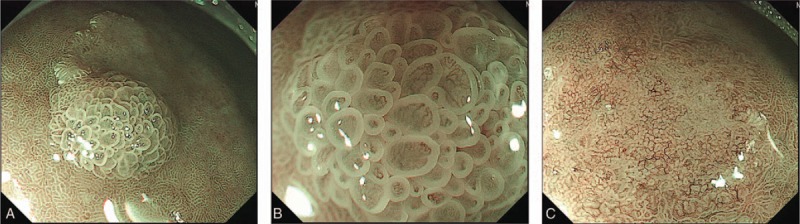
Magnifying endoscopy findings with narrow band imaging. The demarcation line was present around the 0-I + IIa lesion, and the background mucosa of the tumor was regular villi with a light-blue crest (A). Magnifying endoscopy with narrow band imaging of the 0-I lesion revealed irregularly circular marginal crypt epithelium with irregular vessels within the circular intervening part (B). The part of 0-IIa showed a partially absent microsurface pattern partially with white opaque substance and a fine network of irregular microvessels (C).

An 87-year-old man was referred to our institution for endoscopic resection of a gastric lesion. His chief complaint was loss of appetite. There were no significant findings on physical examination. He was positive for *Helicobacter pylori* (Hp) by the urea breath test, and he had never received Hp eradication therapy. An endoscopic examination revealed a reddish, elevated lesion with faint reddish area on the great curvature of the gastric lower body (Fig. 1A and B). The tumor was classified as type 0-I + IIa according to the Paris classification. On magnifying endoscopy with narrow band imaging (ME-NBI), a demarcation line was noted around the 0-I + IIa lesion (Fig. 2A). ME-NBI of the 0-I lesion revealed irregularly circular marginal crypt epithelium with irregular vessels within the circular intervening part (Fig. 2B). The part of 0-IIa showed a partially absent microsurface pattern with white opaque substance and a fine network of irregular microvessels (Fig. 2C). Based on these endoscopic findings, we believed the tumor to be intramucosal gastric adenocarcinoma. We performed endoscopic submucosal dissection (ESD), and the tumor was successfully removed en bloc.

**Figure 3:**
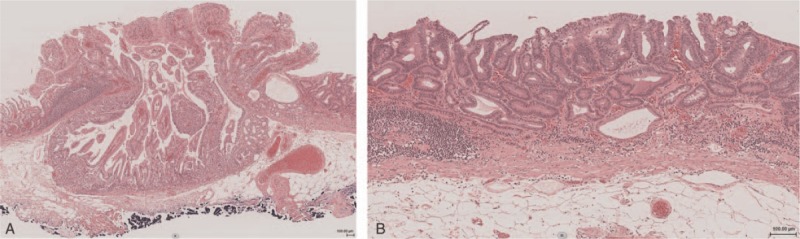
Histological findings. The 0-I lesion was gastric adenocarcinoma composed of columnar cells with low-grade atypia, 3 mm in size (A). The superficial part of the 0-I lesion consisted of a papillary structure mimicking foveolar epithelium with upward growth. The deeper part consisted of a tubular structure resembling the fundic gland showing inverted downward growth to the submucosal layer with the lamina muscularis mucosae. The 0-IIa lesion was well differentiated adenocarcinoma with low-grade atypia, 15 mm in size (B).

The histological findings differed between the 0-I lesion and the 0-IIa lesion. The 0-I lesion was gastric intestinal-type adenocarcinoma (Fig. 3A). The superficial part of the 0-I lesion consisted of a papillary structure mimicking foveolar epithelium with upward growth. The deeper part consisted of a tubular structure resembling the fundic gland that showed inverted downward growth to the submucosal layer with the lamina muscularis mucosae. The 0-I lesion was diagnosed as papillary adenocarcinoma-well-differentiated tubular adenocarcinoma. The 0-IIa lesion was composed of a tubular structure and diagnosed as well differentiated adenocarcinoma with low-grade atypia (Fig. 3B). The 0-I + IIa lesion showed no submucosal or lymphovascular invasion.

**Figure 4:**
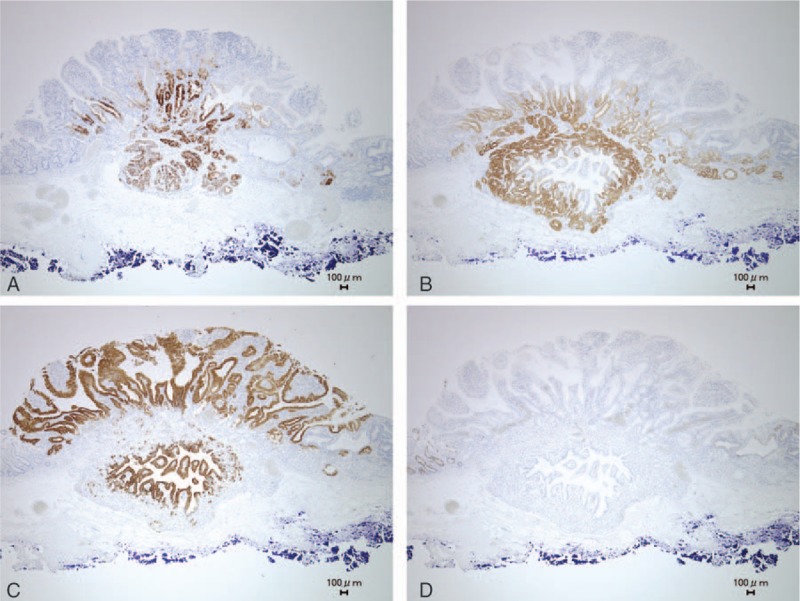
Immunohistological findings. Immunohistochemistry of 0-I the lesion was positive for pepsinogen-I (A), MUC6 (B), and MUC5AC (C) and negative for MUC2 (D). The superficial part of the 0-I lesion consisted of a papillary structure positive for MU5AC that was differentiated to the foveolar epithelium. The deeper part of the 0-I lesion consisted of a tubular structure positive for pepsinogen-I and MUC6 that was differentiated to the fundic gland (chief cells and mucous neck cells).

Immunohistochemistry of the 0-I lesion was positive for Pep, MUC6, and MUC5AC and negative for MUC2 and H+/K^+^ ATPase (Fig. 4A–D). The superficial part of the 0-I lesion was composed of a papillary structure positive for MUC5AC, which had differentiated to foveolar epithelium. The deeper part of the 0-I lesion was composed of a tubular structure positive for Pep and MUC6, which had differentiated to fundic gland (chief cells and mucous neck cells). Therefore, the 0-I lesion was diagnosed as gastric phenotype of adenocarcinoma differentiated to fundic gland mucosa with upward growth in the superficial part and downward growth in the deeper part. The 0-IIa lesion was positive for MUC2 and negative for MUC5AC, and thus the tumor cells of 0-IIa were deemed intestinal phenotype of well differentiated adenocarcinoma.

**Figure 5:**
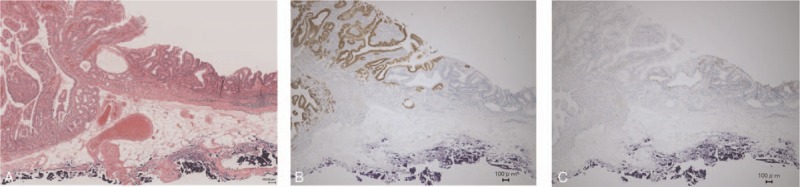
Immunohistological findings. Hematoxylin-eosin staining (A) and immunostaining showed the clear boundary and no transitional tissue between the 0-I and 0-IIa lesions. The 0-I lesion was positive for MUC5AC (B) and negative for MUC2. The 0-IIa lesion was positive for MUC2 (C) and negative for MUC5AC.

The boundary was clear, and no transitional tissue was observed between the 0-I and 0-IIa lesions (Fig. 5A–C), suggesting that the 0-I + IIa lesion was a gastric collision tumor of GA-FGM and well differentiated adenocarcinoma. After ESD, there appeared no complications, such as delayed bleeding or perforation. The patient was discharged 7 days after the treatment. An endoscopic examination at 6 months after ESD revealed no local recurrence.

Ethical approval was not required for this case report as it did not relate to the patient's privacy or treatment. Informed consent for the publication of this case report has obtained.

## 3 Discussion

This is the first report of inverted GA-FGM colliding with well differentiated adenocarcinoma. Two different components led to the characteristic findings (0-I + IIa) on white-light endoscopy. The area of the inverted GA-FGM showed a sessile lesion (0-I), suggesting that inverted GA-FGM exhibits elevation due to the tumor volume on the deeper side. ME-NBI revealed different structures of crypts and vessels among the components, illustrating the collision of 2 types of gastric cancer. ME-NBI also detected irregularly circular marginal crypt epithelium with irregular vessels within the circular intervening part at the 0-I area, which histologically corresponds to a papillary structure mimicking foveolar epithelium at the GA-FGM part, suggesting that ME-NBI can be used to diagnose GA-FGM even if the lesion collides with other types of adenocarcinoma.

Including the present case, only 9 cases of GA-FGMs were reported from 2010 to 2016^[7,8]^ (Table 1). These included 6 male and 3 female patients (mean age, 68.6 years). Five cases were negative for Hp infection, whereas 1 case was positive. Most lesions were detected in the upper third of the stomach. The median size of the lesions was 6 mm. According to the Paris classification, 6 lesions were morphologically classified as nonpolypoid type without mixed type (0-IIa or 0-IIc) and 3 lesions as mixed type (0-IIa + IIc, 0-I + IIa). Three lesions were diagnosed as gastric cancer based on the features of the vessels and crypt structure using ME-NBI.^[9]^ Previous reports indicated difficulty in diagnosing GA-FG by endoscopy, because the tumor cells of GA-FG develop from the deep layer of the lamina propria, which is covered with normal foveolar epithelium.^[4,5]^ However, in our case, the tumor cells of GA-FGM were exposed on the surface of the epithelia, thereby allowing for a diagnosis by ME-NBI.^[8]^ Eight lesions showed submucosal invasion, and only our case was intramucosal cancer with inverted downward growth to the submucosal layer. Immunohistochemically, 1 lesion was predominantly positive for Pep (chief cell dominant type), 2 cases were predominantly positive for MUC6 (mucous neck cell dominant type), and 4 lesions were equally stained for Pep and MUC6 (chief mucous neck combination type). Although GA-FG has been reported to be mainly chief cell-dominant type,^[1]^ GA-FGM appears to include various types of fundic gland differentiation.

**Table 1:**
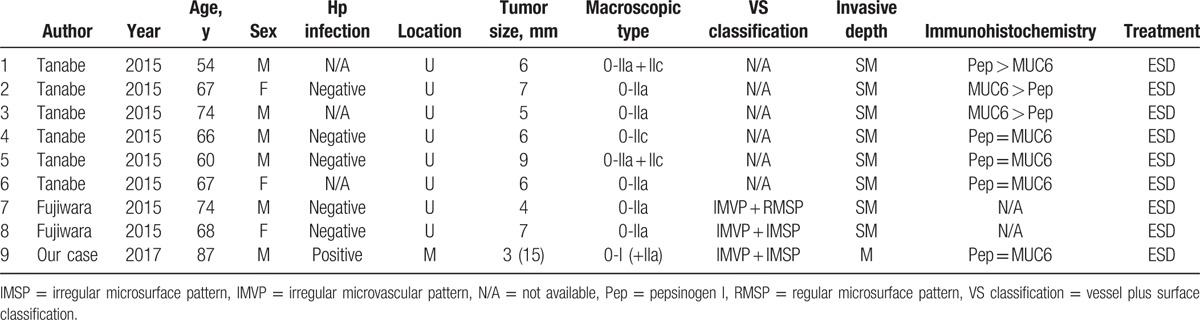
Reported cases of GA-FGM.

The present case exhibited inverted growth that was thought to be due to the downward growth of GA-FGM. Concerning the inverted growth of gastric lesions, such as gastritis cystica profunda, heterotopic mucosa-related carcinoma, and gastric inverted hyperplastic polyp, injury of the muscularis mucosae caused by chronic gastritis and erosion is thought to lead to inversion into the submucosal layer.^[10–12]^ The accumulation of similar cases will help clarify the etiology of the inverted growth of gastric cancers.

## Acknowledgments

No other person contributed to the manuscript.
